# Transcriptomic signatures of hippocampal active place avoidance memory maintenance

**DOI:** 10.3389/fncel.2026.1769317

**Published:** 2026-05-18

**Authors:** Isaac Vingan, Shwetha Phatarpekar, Victoria Sook Keng Tung, Alejandro Ivan Hernández, Oleg V. Evgrafov, Juan Marcos Alarcon

**Affiliations:** 1 School of Graduates Studies, Program in Neural and Behavioral Sciences, State University of New York, Downstate Health Sciences University, Brooklyn, NY, United States; 2 Institute of Genomics in Health, State University of New York, Downstate Health Sciences University, Brooklyn, NY, United States; 3 School of Graduates Studies, Program in Molecular and Cell Biology, State University of New York, Downstate Health Sciences University, Brooklyn, NY, United States; 4 Department of Pathology, State University of New York, Downstate Health Sciences University, Brooklyn, NY, United States; 5 The Robert F. Furchgott Center for Neural & Behavioral Science, State University of New York, Downstate Health Sciences University, Brooklyn, NY, United States; 6 Department of Cell Biology, State University of New York, Downstate Health Sciences University, Brooklyn, NY, United States; 7 Department of Genetics, Human Genetics Institute of New Jersey, Rutgers University, Piscataway, NJ, United States

**Keywords:** gene expression, hippocampus, IEG-*in vivo* tagging, memory, memory-associated neuronal ensemble, offline maintenance, snRNA-seq, spatial transcriptomics

## Abstract

The gene expression changes associated with memory acquisition, consolidation and reconsolidation–all active epochs in memory formation–have been well characterized in the rodent hippocampus. Less is known, however, of the changes in gene expression during the offline maintenance of memory. In this study, we measured the gene expression changes in the dorsal hippocampus of four mice 3 days after consolidation of an active place avoidance memory. We examined gene expression changes in a putative subset of memory-associated neurons by leveraging the immediate early gene *in vivo* tagging system of the Arc-Cre/flox-eYFP transgenic mouse line. Through spatial transcriptomics we found that memory trained animals exhibited spatially regionalized expression of genes involved in post-synaptic function in CA1, synaptic vesicle transport in CA3, and neuronal differentiation in DG. Surprisingly, these gene expression enrichments were not observed in eYFP mRNA positive spatial spots. To gain granularity into this finding, we carried out single nuclear RNA sequencing, which confirmed enrichment of differentially expressed genes associated with synaptic plasticity and post-synaptic signaling unique to each subregion in trained animals, but not from their eYFP mRNA positive nuclei. Notably, nuclei of hippocampal neurons were largely characterized by their down regulation of genes involved in ATP synthesis and cytoplasmic translation. Our results suggest that two overarching transcriptomic patterns contribute to the functional changes in hippocampal cells during offline memory maintenance: regionally distributed expression of genes linked to synaptic functions (with concomitant sparseness of memory-associated neuronal ensembles) and a reduction of metabolic activity related genes across hippocampal sub-regions.

## 1 Introduction

The diversity of transcriptomic profiles across brain systems is thought to be essential for the functioning of neuronal ensembles that support memory (Cembrowski et al., 2016a; Cembrowski et al., 2016b; Marco et al., 2020; Rao-Ruiz et al., 2019a; Yao et al., 2021). This idea is grounded in studies demonstrating that the acquisition, recall, and long-term persistence of learned information depend on synaptic plasticity mechanisms expressed at the synapses connecting these ensembles (Kitamura et al., 2017; Josselyn and Tonegawa, 2020; Mayford, 2014). Moreover, gene expression changes are well established as a critical requirement for supporting activity-dependent synaptic plasticity (Rao-Ruiz et al., 2019a; Shpokayte et al., 2022; Sullivan et al., 2021; Shah et al., 2016; Cho et al., 2016).

Our laboratory recently identified distinct transcriptomic signatures associated with the consolidation of an active place avoidance (APA) memory across subregions of the dorsal hippocampus (Vingan et al., 2024). We found that the expression of these signatures correlated with neuronal populations defined by their expression of individual immediate-early genes (IEGs)—Arc, Egr1, or c-Jun—suggesting a specific transcriptomic architecture underlying memory consolidation. Memory-associated neuronal ensembles are proposed to reorganize across different phases of memory processing, including acquisition, reactivation, and storage (Guan et al., 2016; Chen et al., 2019; Senzai, 2019; Kesner et al., 2004; Lisman, 1999). In the present study, we sought to determine whether such reorganization involves distinct transcriptomic profiles for memory consolidation versus memory maintenance.

To address this question, we collected hippocampal tissue for spatial transcriptomics and single-nucleus RNA sequencing from mice trained in an APA memory task—or from untrained controls—3 days after a post-training memory retention test, and critically, without experimentally triggering memory recall. To capture the neuronal populations active during consolidation, we used Arc-Cre/flox-eYFP transgenic mice (Denny et al., 2014) to permanently label neurons that expressed the activity-dependent IEG Arc during the retention test. By examining gene-expression changes across hippocampal regions and within tagged neuronal populations 3 days later, we aim to identify transcriptomic shifts associated with the offline maintenance of a stabilized memory trace.

## 2 Results

### 2.1 Animals trained in APA show robust post-training memory retention test

**Figure 1:**
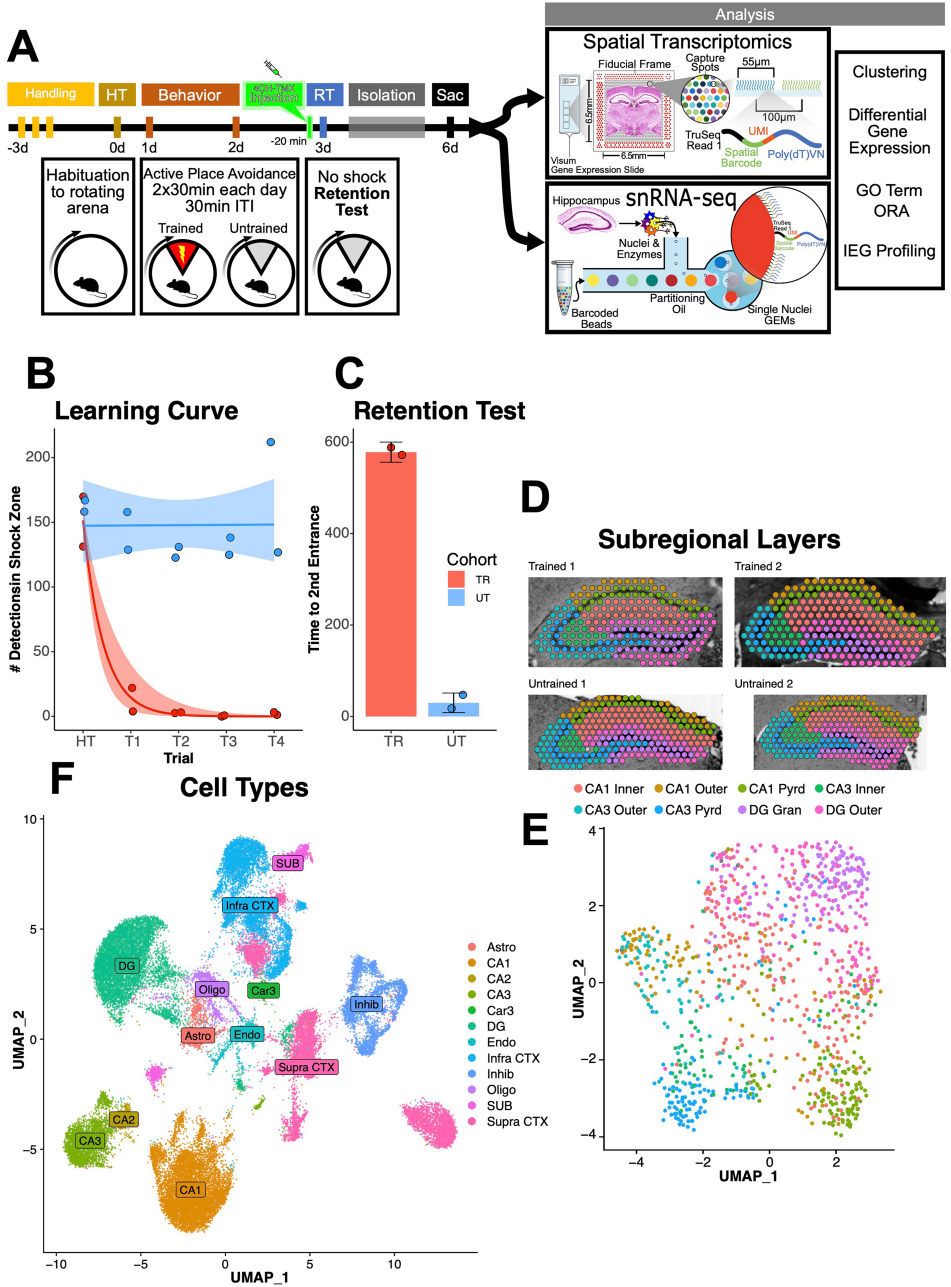
APA training and spatial transcriptomics mapping of the mouse hippocampus. **(A)** Experimental timeline. Mice were trained in the APA paradigm over the course of 4 days following 3 days of handling. On day 0 mice were habituated to a rotating arena with no active shock zone (HT). On days 1 and 2 mice assigned to the trained behavioral condition (*n* = 2, male) received two 30-min trials with an active shock zone. Mice assigned to the untrained behavioral condition (*n* = 2, male) were not exposed to an active shock zone. On day 3, all mice received an IP injection of 4-hydroxytamoxifen to trigger permissive tagging of Arc-expressing neurons with eYFP. Thirty minutes post injection, mice received a 10-min retention test trial (exposed to the rotating arena with no active shock zone). Following the retention test, mice were returned to the colony room and single housed in a sound attenuation chamber for 3 days to limit stimulation and spurious eYFP neuronal tagging. Mice were sacrificed at end of this 3-day interval and brains were collected for further processing. **(B)** Training performance measured as number detections in the shock zone. Mice assigned to the trained behavioral condition learned to avoid the location of the shock zone indicated by the decreased number of shocked triggered. Exponential fit learning curves included with shaded error bars (SE). **(C)** Memory performance measured as time to second entrance in the retention test trial. Trained mice demonstrated higher time to second entrance during the retention test. Line and point color reflect the assigned behavioral condition (untrained = blue, trained = red). **(D,E)** Spatial transcriptomics map **(D)** and UMAP plot of cell-layer annotated spatial transcriptomic maps of hippocampal spots **(E)** in samples from trained and untrained mice. Discrete color scale reflects the predominant anatomical cell layer surveyed by each capture spot which was determined through assistance by query-based cell-type annotation through data integration with the single cell RNA-seq Allen Brain Atlas cortex and hippocampus in mice. **(F)** UMAP plot of cell-type annotated hippocampal nuclei clusters. Discrete color scale reflects the predominant cell type surveyed by each cluster which was determined through assistance by query-based cell-type annotation through data integration with the single cell RNA-seq Allen Brain Atlas cortex and hippocampus in mice.

To investigate gene-expression changes associated with offline memory maintenance in the hippocampal network, we performed spatial transcriptomics and single-nucleus RNA sequencing (snRNA-seq) 3 days after an active place avoidance (APA) memory-retention test using Arc-Cre/flox-eYFP mice (Figure 1A). After intraperitoneal injection of 4-hydroxytamoxifen, Arc-expressing neurons in Arc-Cre/flox-eYFP mice permanently express the fluorescent reporter eYFP (Denny et al., 2014).

The APA task requires mice to learn to avoid a 60° shock zone on a rotating circular arena (Cimadevilla et al., 2000; Stuchlik et al., 2013). APA learning occurs rapidly, and the task variant used here produces avoidance memory that persists for at least 1 month (Pavlowsky et al., 2017; Tsokas et al., 2016). APA-trained mice (*n* = 2 males) showed fewer shock-zone detections during acquisition compared to untrained mice (*n* = 2 males), which were exposed to the same apparatus with the shock disabled (Figure 1B). One day later, trained mice exhibited longer shock zone re-entry latencies during the memory retention test compared to untrained mice (Figure 1C). Re-entry times (i.e., time to second entrance) served as the retention test performance metric because it is less sensitive to initial self-localizations errors when the mouse is place into the rotating arena (Cimadevilla et al., 2000; Stuchlik et al., 2013; Cimadevilla et al., 2001). After the retention test, animals were returned to their home cages and housed inside a sound attenuation chamber. Three days later, mice were removed from their cages and immediately euthanized for brain collection and flash-freezing. Hippocampal tissue from all animals were used for *both* spatial transcriptomic and snRNA-seq studies.

### 2.2 Spatial transcriptomics demonstrates few DEGs identified amongst eYFP-expressing spots 3 days after APA memory post-training retention test

**Figure 2:**
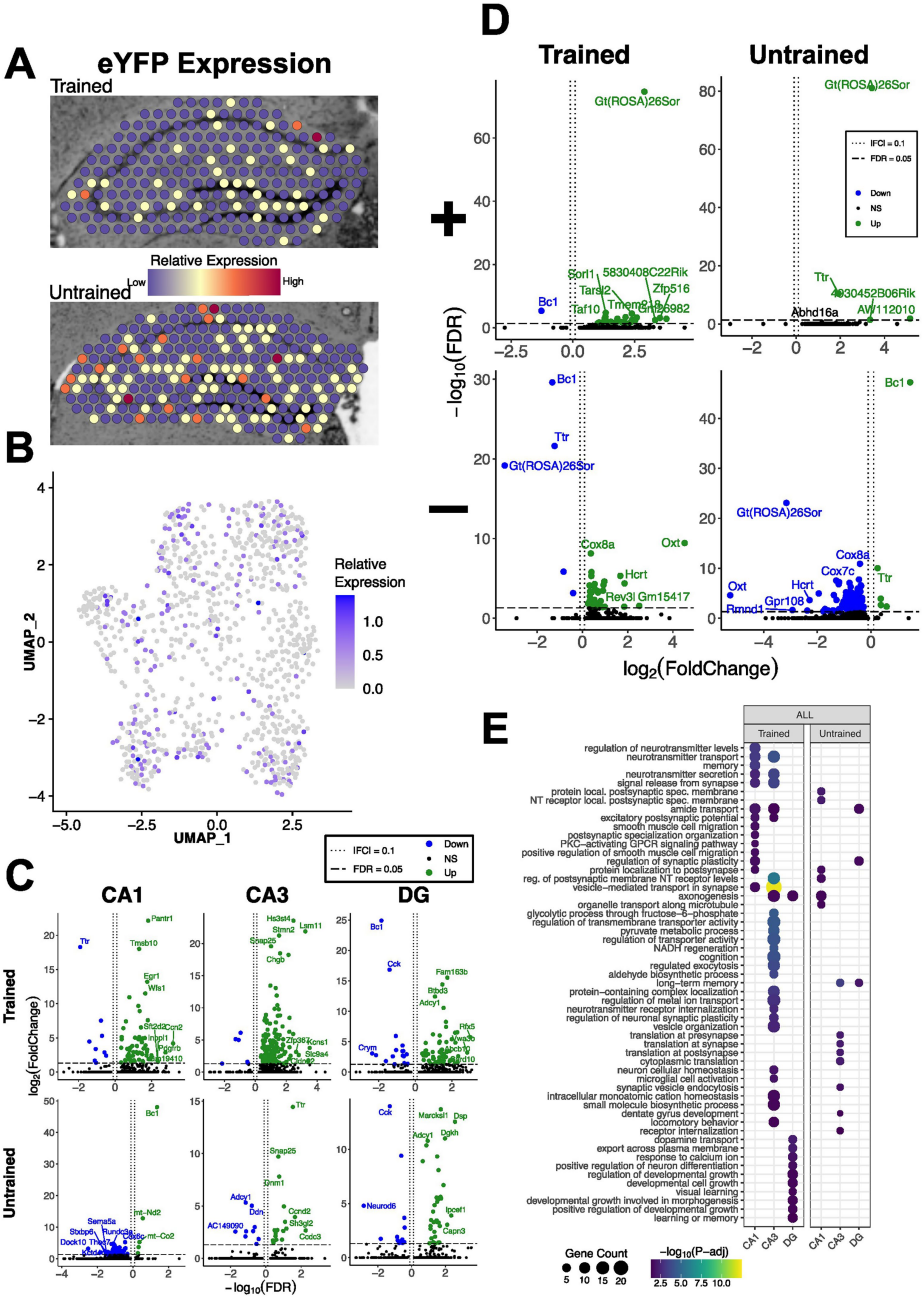
APA memory maintenance specific genes and their association with eYFP-positive spots in the hippocampus. **(A,B)** Representative spatial transcriptomic maps **(A)** UMAP plot of hippocampal spots **(B)** in the trained (top) and untrained (bottom) samples colored to reflect relative expression of the eYFP mRNA transcript, Gt(ROSA)26Sor. **(C)** Volcano plot of DEGs detected in the combined analysis of hippocampal spots across trained and untrained conditions. In the trained condition, 70 DEGs were upregulated and 9 DEGs were downregulated in the CA1 (top left), 143 and 6 in the CA3 (top middle), and 59 and 17 in the DG (top right). In the untrained condition, 5 DEGs were upregulated and 56 DEGs were downregulated in the CA1 (bottom left), 18 and 9 in the CA3 (bottom middle), and 34 and 13 in the DG (bottom right). **(D)** Volcano plots of differential gene expression of eYFP positive and negative spots in the trained and untrained conditions. In the analysis of eYFP*+* spots, in the trained condition (top left) 26 upregulated and 1 downregulated gene were detected, and in the untrained condition (top right) 4 upregulated and 0 downregulated genes were detected. In the analysis of eYFP*-* spots in the trained condition (bottom left) 35 upregulated and 5 downregulated genes were detected, and in the untrained condition (bottom right) 5 upregulated and 155 downregulated genes were detected. **(E)** Regional enrichment of biological processes detected amongst all differentially expressed (stratification of analysis by up- and down-regulated genes can be found in Supplementary Figure 2). Dot color reflects the statistical significance (−log_10_(FDR)) of the biological process enrichment. Dot size reflects the number of detected DEGs mapped to the genes involved in a given biological process.

To investigate the spatial distribution of differentially expressed genes (DEGs) associated with offline maintenance of APA memory across the hippocampus, we performed spatial transcriptomics on coronal sections containing the dorsal hippocampus from the two trained and the two untrained mice. We performed clustering on the integrated spatial transcriptomic dataset, identifying distinct clusters that mapped onto anatomical boundaries as visualized in both spatial and UMAP plots (Figure 1D). We integrated the spatial transcriptomic data with the Allen Brain Atlas (Yao et al., 2021) to generate semi-supervised cell-type annotations for each capture spot (Figure 1E). We then examined anatomically defined hippocampal subregions for memory-associated differences in gene expression. Differential gene expression analysis between trained and untrained mice identified total 129 DEGs in all hippocampal spots. Stratification by subregions detected 79 DEGs in the CA1, 149 in the CA3, and 76 in the DG in the trained condition. In the untrained condition, we detected 61 DEGs in the CA1, 27 in the CA3, and 47 in the DG (Figure 2C). Gene ontology term (GO-term) enrichment analyses of the DEGs detected between the hippocampal spots of the trained and untrained cohorts revealed overrepresentation of biological processes related to ATP production and ribosomal assembly in the trained condition (Supplementary Figure 2). DEGs detected in the analysis of the CA1 spots in the trained condition were enriched with genes involved in biological processes related to post synaptic function. In the CA3 of the trained condition, DEGs were enriched with genes involved in glycolysis and synaptic vesicle transport. And in the DG of the trained condition, downregulated DEGs were enriched with genes involved in neurite growth and neuronal differentiation.

Next, we leveraged the Arc-Cre/flox-eYFP double transgenic mouse line to tag Arc-expressing memory-associated neuronal populations which were identified by the detection of eYFP-expression in spatial transcriptomic spots. Of 460 hippocampal spots from trained mice, 79 spots (~17%) showed detectable expression of Gt(ROSA)26Sor mRNA (eYFP+). In the untrained animals, we detected 77 eYFP+ spots (~14%) out of 530 total hippocampal spots (Figures 2A,B). Regionally, CA1 contained the highest number of eYFP+ spots in both trained and untrained cohorts. We then performed differential expression testing amongst groups of eYFP positive and negative hippocampal spots in each training cohort (Figure 2D). We identified 27 DEGs among eYFP+ spots in trained mice and 4 in untrained mice. GO-term analyses did not identify significantly enrichened biological processes, likely due to the small number of DEGs in the eYFP+ spots. Nonetheless, GO-term analyses across hippocampal regions of trained mice revealed enrichment of biological processes related to synaptic transmission (CA1), energy metabolism (CA3), and neuronal growth (DG) (Figure 2E).

### 2.3 snRNA-seq detects few DEGs amongst sub-regional populations of eYFP-expressing nuclei 3 days after APA memory post-training retention test

**Figure 3:**
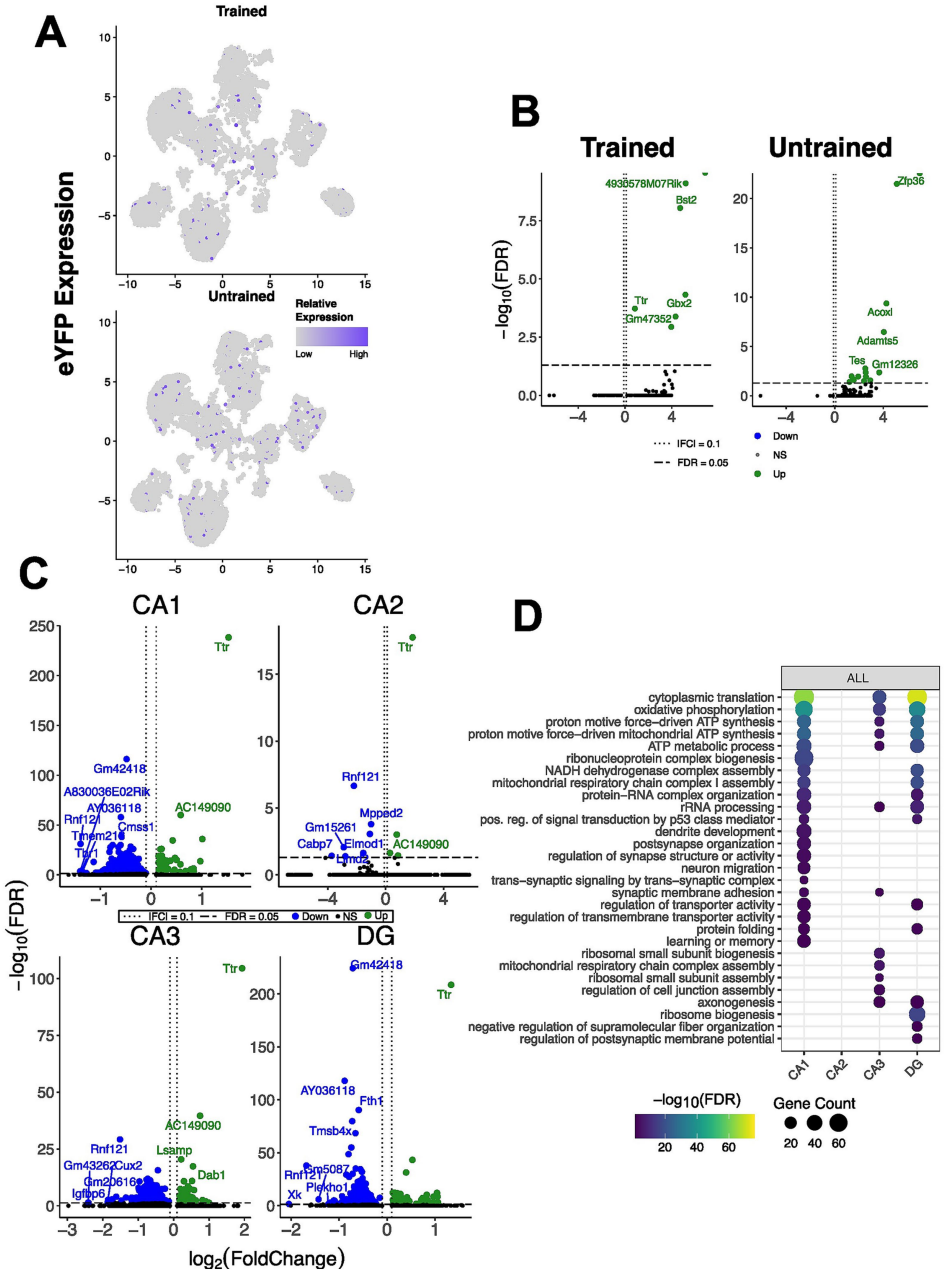
snRNAseq confirms hippocampal regionalization of memory-associated DEGs and few DEGs in eYFP-positive nuclei, and reveals an overall downregulation of ATP metabolism and cytoplasmic translation in APA memory maintenance. **(A)** UMAP plot of hippocampal nuclei split by behavioral conditions showing eYFP+ nuclei in each cluster. Datapoints are colored with a continuous color scale to reflect the relative expression of the eYFP mRNA transcript, Gt(ROSA)26Sor. **(B)** Volcano plots of differential gene expression in eYFP+ nuclei in the trained (left) and untrained (right) conditions (volcano plots of regionally stratified analysis of eYFP+ nuclei in Supplementary Figure 6). **(C)** Volcano plot of DEGs between nuclei derived from trained and untrained animals for each cluster of annotated hippocampal nuclei. In the analysis of CA1 nuclei comparing trained and untrained conditions (top left), 54 genes were upregulated and 775 were downregulated in the trained condition. Four genes were upregulated and 7 were downregulated in the CA2 nuclei (top right), 45 genes were upregulated and 267 were downregulated in the CA3 nuclei (bottom left), and 65 genes were upregulated and 464 were downregulated in the DG nuclei (bottom right). **(D)** Regional enrichment of biological processes detected amongst all DEGs (stratification by up- and down-regulated genes can be found in Supplementary Figure 4). Dot color reflects the statistical significance (−log_10_(FDR)) of the biological process enrichment. Dot size reflects the number of detected DEGs mapped to the genes involved in a given biological process.

To characterize cell-type–specific transcriptional changes, we performed snRNA-seq on nuclei isolated from hippocampal tissue of the same trained and untrained animals used for spatial transcriptomics. We examined the eYFP-expressing population of hippocampal nuclei to assess the changes in gene expression amongst the hippocampal cells putatively associated with the active place avoidance memory. In total, we detected 785 eYFP+ nuclei: 247 from the trained mice and 538 eYFP+ from the untrained mice (Figure 3A). The highest proportion of eYFP+ nuclei was mapped to the CA1 cell type cluster for both groups.

We first compared all eYFP+ nuclei between trained and untrained mice, identifying 7 DEGs in trained animals and 14 in untrained animals. When stratified by hippocampal region, we detected 83, 161, 105, and 83 DEGs in CA1, CA2, CA3, and DG of trained animals, respectively (Figure 3B; Supplementary Figure 6). In untrained animals, we detected 83, 325, 261, and 161 DEGs in the CA1, CA2, CA3, DG, respectively (Figure 3B; Supplementary Figure 6). As in the spatial transcriptomics dataset, GO-term analyses did not reveal significant enrichment, likely reflecting limited DEG sets within eYFP+ nuclei.

### 2.4 snRNA-seq of regionally distinct hippocampal nuclei reveals differential expression of memory-associated

To investigate regionally distinct hippocampal cells, sequenced nuclei were clustered and annotated with cell types through query based annotation and integration with a subset of the single cell Allen Brain Atlas for Cortex and Hippocampus (Figure 1F) (Yao et al., 2021). We analyzed a total of 45,700 nuclei (16,971 from the trained animals, 28,729 from the untrained animals), identifying 12 major cell type clusters including CA1–3, DG, subiculum, inhibitory neurons, cortical neurons, astrocytes, oligodendrocytes, endothelial cells and Car3 neurons.

Next, we performed differential gene expression analyses to identify the genes and delineate molecular functions that are differentially regulated in the cells of the hippocampus. We performed differential-expression analyses within each cluster to identify genes and pathways regulated by prior APA training. We identified 829 DEGs in CA1, 11 in CA2, 312 in CA3, and 529 in the DG (Figure 3C). Inhibitory neurons, astrocytes, and oligodendrocytes showed 275, 54, and 100 DEGs, respectively (Figure 3C). GO-term analyses revealed enrichment of biological processes related to learning and memory (CA1), mRNA processing (CA2), cell–cell adhesion (CA3), and synaptic signaling (DG) (Figure 3D). Notably, downregulated DEGs in CA1, CA3, and DG were enriched for processes related to cytoplasmic translation and metabolism. Similarly, inhibitory neurons from trained mice showed downregulation of genes involved in cytoplasmic translation and ATP production (Supplementary Figure 5). Glial populations exhibited enrichment for biological processes overlapping with those identified in excitatory hippocampal nuclei.

## 3 Discussion

We utilized spatial transcriptomics and snRNA-seq in an integrative investigation of the hippocampal transcriptional architecture associated with offline memory maintenance 3 days after the post-training retention test of an APA task. Unexpectedly, we detected relatively few DEGs within the putative APA memory associated neurons, identified by their persistent expression of eYFP mRNA. Spatial transcriptomics revealed that CA1, CA3 and DG subregions were, respectively, characterized by gene expression signatures related to postsynaptic function, synaptic vesicle transport, and neuronal differentiation. snRNA-seq further showed that neuronal nuclei across the hippocampus were broadly defined by their downregulation genes involved in ATP synthesis and cytoplasmic translation.

### 3.1 Few molecular features define the memory associated eYFP expressing neuronal ensembles in memory maintenance

We leveraged the Arc-Cre/eYFP tagging strategy to examine transcriptomic changes in neurons strongly activated during the APA retention test—the putative memory-associated ensemble. Although IEG-based tagging systems are widely used to study memory-associated ensembles, relatively few studies have examined their transcriptomic signatures (He et al., 2019; Minatohara et al., 2015; Nambu et al., 2022; Fujihara et al., 2009; Denny et al., 2014; Sun and Lin, 2016), but few studies have focused on the transcriptomic changes defining this critical population of neurons (Gonzales et al., 2020; Jaeger et al., 2018; Wu et al., 2017; Butto et al., 2024; Cho et al., 2016; Sullivan et al., 2021; Fuentes-Ramos et al., 2021; Vanrobaeys et al., 2023; Rao-Ruiz et al., 2019a).

Spatial transcriptomics revealed upregulation of *Dr1* and *Gad2* in eYFP positive spots, suggesting contributions of dopaminergic signaling and inhibitory modulation to the memory-related microenvironments (Rossato et al., 2009; Topolnik and Tamboli, 2022). snRNA-seq further identified upregulation of *Prkcb* in the CA1 which has been shown to enhance synaptic efficacy in memory, and *Grik4* in the CA3 which has been shown to regulate synaptic inhibition in memory (Sun and Alkon, 2014; Pressey and Woodin, 2021). Differential expression testing also revealed distinct sets of genes marking eYFP positive nuclei from each hippocampal subregion in the trained mouse. These results indicate that transcriptomic mechanisms supporting memory maintenance in the eYFP-positive neurons vary by hippocampal subregion. We propose that the sparsity of DEGs reflects engrammatic pruning that occurs as consolidated memories enter an offline state (Han et al., 2007; Guan et al., 2016; Rao-Ruiz et al., 2019b) consistent with prior reports of ensemble contraction following memory acquisition (Denny et al., 2014; Tome et al., 2024; Sweis et al., 2021).

### 3.2 Memory maintenance is supported by subregional gene expression profiles and metabolic dampening in the hippocampus

Most molecular studies of memory focus on acquisition, recall, and retrieval (Frankland et al., 2019; Szapiro et al., 2002; Asok et al., 2019; Tronson and Taylor, 2007; Albo and Graff, 2018; Hsieh et al., 2017; Chen et al., 2020; Zovkic et al., 2013; Sun et al., 2024). These phases involved bursts of new protein synthesis and dynamic redistribution of the memory trace across neuronal systems (Blum et al., 2006; Dudai et al., 2015; Albo and Graff, 2018; Kim et al., 2018; Haubrich et al., 2020; Chen et al., 2020). In contrast, the neuronal processes underlying offline memory maintenance remain poorly understood (Asok et al., 2019; Sheng and Kim, 2011; Guedes-Dias and Holzbaur, 2019; Alabi and Tsien, 2012). Through spatial transcriptomics, we observed enrichment of postsynaptic specialization genes in CA1 and synaptic-vesicle–related genes in CA3; evidence that suggests for a regionalized role of cell transcriptomes in memory maintenance. snRNA-seq confirmed regionalized transcriptional features but revealed a dominant pattern of downregulated genes involved in ATP synthesis and translation. This pattern may reflect an unrecognized role for energy conservation and reduced protein synthesis during offline memory maintenance (Li and Sheng, 2022; Kann, 2016). A key technical caveat is the known mismatch between nuclear and whole-cell transcriptomes (Ding et al., 2020; Oh et al., 2022; Bakken et al., 2018). Together, our data suggest that although hippocampal subregions remain transcriptionally engaged in processes modulating excitability, hippocampal neurons broadly adopt a metabolically and translationally suppressed state during offline maintenance.

### 3.3 Technical limitations

In this study, brief chemical restraint (rather than physical restraint) was selected to enable the slow intraperitoneal administration of a viscous 4-hydroxytamoxifen suspension and minimize stress-induced transcriptional changes like those caused by physical restraint that could obscure the learning-specific signals we aimed to capture. However, we must consider that the use of Isoflurane anesthesia in this experiment potentially confound the interpretation of our results. Fluorinated gas anesthetics have been shown to alter neuronal activity and gene expression following prolonged exposures (20 min to several hours) (Pekny et al., 2014; Wang et al., 2020); the effects of brief exposures, like those used in our experiment, are less characterized. In contrast to brief chemical restraint, physical restraint is known to induces robust acute stress responses that activate immediate early genes and stress-related transcriptional programs that would directly confound our measurement of learning-specific molecular signatures (Waag et al., 2025).

Importantly, both trained and untrained control animals underwent identical anesthesia exposure and 4-hydroxytamoxifen administration, such that all transcriptomic comparisons presented here reflect differences between training conditions under equivalent pharmacological and anesthetic backgrounds. While we cannot definitively exclude a transient contribution of anesthesia to the observed gene expression patterns, the robust behavioral performance during retention suggests effective memory retrieval and supports the interpretation that Arc-positive neurons reflect memory reactivation (Josselyn et al., 2015).

Our findings must also be interpreted considering limitations inherent to the transcriptomic approaches used. Tissue for both modalities was collected from the same tissue block to fully utilize our limited sample size of four mice (two trained, two untrained). 10-μm sections collected for spatial transcriptomic sections represent a single rostral cross section of the hippocampus while tissues used for snRNA-seq were collected from a wider array of hippocampal loci. Anatomical differences along the hippocampal dorsal-ventral axis could themselves account for transcriptional differences between the two datasets (Cembrowski et al., 2016b). Additionally, spatial transcriptomics samples *in situ* microenvironments using capture spots larger than individual neuronal somata (Duan et al., 2023; Rao et al., 2021), whereas snRNA-seq isolates nuclear mRNA and excludes transcripts previously trafficked to the soma or projections (Ding et al., 2020; Lacar et al., 2016; Thrupp et al., 2020). Thus, the spatial transcriptomic data reflect the cumulative effects of memory maintenance across local microenvironments, while the snRNA-seq data capture nuclear-localized transcriptional states at the time of collection.

### 3.4 Transcriptomic signatures of memory maintenance may reflect contraction of the memory trace as part of systems consolidation

Offline memory maintenance occupies the transitional period between consolidation—which involves LTP and protein synthesis (Rosenberg et al., 2014; Sacktor, 2008; Lynch, 2004; Hernandez and Abel, 2008; Jarome and Helmstetter, 2014)—and remote storage, which reflects distributed systems-level memory organization (Albo and Graff, 2018; Doron and Goshen, 2018). Compellingly, Cho et al. demonstrated that transcriptional repression up to 4 h after contextual fear conditioning in mice was critical for memory retention and was driven by estrogen receptor 1 signaling (Cho et al., 2015). Such transcriptional repression in the hippocampus could be a persistent mechanism–up to 72 h, as with the animals in this study–that reduces background transcriptional activity and primes the neuron for new epochs of transcriptional demand and memory retention.

The paucity of memory-associated DEGs may also reflect ongoing reorganization and sparsing of the hippocampal memory trace during this intermediate phase. The molecular imprint of the memory trace, readily detectable after consolidation, may become less distinguishable during offline maintenance consistent with the idea that the hippocampal trace contracts into a residual index (Goode et al., 2020; Tanaka and McHugh, 2018; Teyler and DiScenna, 1986), transcriptionally muted yet poised for reactivation. Alternatively, in the absence of retrieval, such a reduced index may remain vulnerable to forgetting.

## 4 Methods

### 4.1 Animals

Four total adult male *ArcCreERT2:eYFPflx* mice (C57BL/6 background, 3–4 months old) were used in both the spatial transcriptomics and snRNA-seq studies (two trained, two untrained). Mice were bred in-house at SUNY Downstate Health Sciences University (Brooklyn, NY) and maintained under standard conditions with ad libitum access to food and water. Animals were group-housed (2–5/cage) and singly housed during behavioral testing in sound-attenuation cubicles (Med Associates). Mice were randomly assigned to cohorts and handled for 3 days before experiments to reduce stress. All procedures were approved by the SUNY Downstate Institutional Animal Care and Use Committee.

### 4.2 Behavior

All procedures were performed in compliance with the Institutional Animal Care and Use Committee of the State University of New York, Downstate Health Sciences University. Male *ArcCreERT2:eYFPflx* mice were trained in the hippocampus-dependent two-frame active place avoidance (APA) task. The apparatus (Bio-Signal Group Corp., Brooklyn, NY) consisted of a 40 cm rotating arena (1 rpm) with a parallel-rod floor delivering a scrambled 0.2 mA, 500 ms foot shock (60 Hz) upon entry into a 60° shock zone. Video tracking (30 Hz) monitored animal position, and performance was quantified as shock-zone detections using TrackAnalysis software.

Trained mice received one 30-min habituation trial (shock off) followed by four 30-min training trials over 2 days (shock on, 40 min inter-trial interval). Untrained controls experienced identical sessions without shock. Memory retention was tested 24 h after the final training session in a 10-min trial (shock off), with latency to second zone entry serving as the primary performance metric, a validated measure of memory recall robust to self-localization error (Cimadevilla et al., 2000; Stuchlik et al., 2013; Cimadevilla et al., 2001).

### 4.3 *In vivo* arc-driven neuronal tagging

Identification of neurons strongly activated during memory reactivation is made possible through the use of *Arc-Cre/flox-eYFP* mice (Denny et al., 2014). In this animal model, the *Arc* promoter drives a tamoxifen-inducible Cre recombinase, which activates the floxed *R26R* promoter to induce persistent eYFP expression. Within 20 min following an IP injection of 4-hydroxytamoxifen (1.5 mg/mL), eYFP expression in these mice. IP injections of a 4-hydroxytamoxifen/oil suspension were performed under anesthesia (5% isoflurane vaporized in 100% oxygen). Anesthesia was delivered in a sealed induction chamber for no more than 2 min, or until the animal became unresponsive to physical stimulation. Animals were removed from the induction chamber to perform the controlled IP injection of the viscous suspension. Mice were monitored for the return of the righting reflex before being returned to their home cage to rest for 45 min before beginning the 10-min retention test. Following the retention test mice were returned to their home cage and remained undisturbed in sound-attenuated single housing for 3 days to prevent spurious activation while labeling was active. This system enabled temporally restricted tagging of *Arc*-expressing (eYFP^+^) neuronal ensembles.

### 4.4 Tissue collection for transcriptomic experiments

Three days after the retention test, mice were euthanized, and brains were rapidly extracted, embedded in Tissue-Tek O.C.T. compound (Sakura Finetek, USA), and snap-frozen in methyl-butane chilled by liquid nitrogen. Samples designated for spatial transcriptomics and snRNA-seq processing were collected from the same specimen blocks of the same four mice (two trained, two untrained).

Coronal sections containing the dorsal hippocampus (bregma −1.7 to −2.2 mm) were cut at −20 °C. The specimen block was trimmed in thick sections (50 μm) to reveal visually intact regions of the dorsal hippocampus for thin sectioning (10 μm). Cross sections containing the right hippocampus were extracted from these thick sections and set aside for snRNA-seq (see 6.8). Intact, thin sections were reserved for spatial transcriptomics (see 6.5). After successful collection of a thin section, additional thick sections containing the right dorsal hippocampus were collected, cut, and reserved for snRNA-seq.

### 4.5 Tissue preparation for spatial transcriptomics

Visually intact coronal sections (10 μm) spanning the dorsal hippocampus (bregma −1.7 to −2.2 mm) were cut at −20 °C and mounted on 10x Genomics Visium Gene Expression. Ideal sections from each specimen were cut to fit within one of four 5 × 5 mm capture area, fixed in pre-chilled methanol, and processed using the manufacturer’s “Visium Spatial Gene Expression Reagent Kits User Guide, Document Number CG000239 Rev. H, 10x Genomics, (2024, August 15).”

Tissue permeabilization time was optimized to 18 min, as determined by processing samples according to “Visium Spatial Tissue Optimization Reagents Kits User Guide, Document Number CG000238 Rev. F, 10x Genomics, (2024, August 15).” Brightfield images were captured with a Leica Aperio CS2 slide scanner (20×), and fluorescent images with a Zeiss LSM 800 (555 nm LED, 75% intensity, 200 ms exposure). Libraries were prepared according to the Visium Spatial Gene Expression Reagent Kits User Guide and sequenced on an Illumina NovaSeq 6000 (NovaSeq S4 kit, 200 cycles), targeting ~2 × 10^8^ read pairs per sample (50,000 reads × 80% of 5,000 capture spots). Sequencing was performed using read lengths of 28 bp (read 1), 10 bp (i7), 10 bp (i5), and 91 bp (read 2).

### 4.6 Spatial transcriptomics preprocessing

Raw sequencing data from the NovaSeq 6000 were converted from BCL to FASTQ format and demultiplexed with *bcl2fastq2 v2.20*. Adapter trimming and quality control were performed with *TrimGalore v0.6.5*. Processed reads were aligned and quantified using *Space Ranger v2.0.0* (10x Genomics) against the *mm10 (refdata-gex-mm10-2020-A)* mouse genome. Gene-barcode matrices were generated and normalized to correct technical variation, and spatial coordinates were aligned with histological images to localize transcriptional activity.

### 4.7 Spatial transcriptomics analysis

Gene expression matrices were analyzed in Seurat v5 (R environment) (Hao et al., 2023; Butler et al., 2018). Capture spots were restricted to the dorsal hippocampus. Normalization and variance transformation of RNA-seq data from spots wereperformed using SCTransform v2 (Seurat). After integration and clustering, data were visualized in UMAP and spatial coordinates. Cell-type annotation was performed by integrating our dataset with 10,000 hippocampal cells from the Allen Brain Atlas Cortex and Hippocampus Taxonomy (Yao et al., 2021), assigning identities based on transcriptomic similarity and anatomical position.

Differential expression testing was performed using the Wilcoxon rank-sum test. Spots from the entire hippocampus or individual subregions (CA1, CA3, DG) were compared between trained and untrained groups. Within each condition, spots were further stratified by detectable expression of immediate early genes (e.g., *Arc^+^* vs. *Arc^−^*). Genes with FDR < 0.05 were considered differentially expressed.

We conducted Gene Ontology (GO) enrichment analysis on lists of differentially expressed genes using *clusterProfiler* v4.10.0 in R (Wu et al., 2021), focusing on biological processes, cellular components, and molecular functions. The *clusterProfiler* R package depends on the Bioconductor annotation data ‘GO.db’ and package ‘org. Mm.eg.db’ for genome-wide annotation of mouse genes. Our analyzes utilized a significance threshold of FDR < 0.01 for GO terms. Redundant terms were filtered out using simplify() with a cutoff of 0.7. The top 10 most significant GO terms were displayed in the ranked dot plot.

### 4.8 Nuclei isolation and counting for snRNA-seq

Frozen hippocampi were minced and processed into single-nuclei suspensions using a modified “Daughter of Frankenstein” protocol (Martelotto, 2021). Tissue was homogenized on ice in IGEPAL-40 buffer, filtered (70 μm), and centrifuged (500 × *g*, 4 °C). Nuclei were purified by OptiPrep density gradient centrifugation, resuspended in wash buffer, filtered (40 μm), and stained with trypan blue for counting. Concentrations were determined by hemocytometer, and suspensions were diluted to ~8,000 nuclei in 75 μL for loading.

### 4.9 Droplet-based nuclei separation

Single-nucleus cDNA libraries were generated according to the “Chromium Single Cell 3’ Reagent Kits User Guide (v3.1 Chemistry) User Guide, Document Number CG000204 Rev. D, 10x Genomics, (2022, July 12).” using the 10x Genomics Chromium Single Cell 3′ kit (protocol CG000204). Nuclei, partitioning oil, and barcoded gel beads were co-encapsulated in droplets, followed by reverse transcription and library preparation per manufacturer’s instructions. Libraries were sequenced on an Illumina NovaSeq 6000 (NovaSeq S4 kit, 200 cycles), targeting ~20,000 reads per nucleus. Read configuration: 28 bp (read 1), 10 bp (i7), 10 bp (i5), 91 bp (read 2).

### 4.10 Single nuclei RNA SEQ processing

Raw NovaSeq data were converted to FASTQ and demultiplexed (*bcl2fastq2 v2.20*), trimmed (*TrimGalore v0.6.5*), and processed with *Cell Ranger v8.0* (10x Genomics) for alignment, quantification, and normalization against the *mm10 (refdata-gex-mm10-2020-A)* genome. Gene-barcode matrices were generated and normalized to account for technical variability.

### 4.11 snRNA-SEQ analysis

Processed matrices were analyzed in Seurat v5. Low-quality barcodes (<500 detected genes or >5% mitochondrial transcripts) and doublets [identified with *DoubletFinder* (McGinnis et al., 2019)] were excluded. Normalization and variance stabilization of cleaned datasets were performed usingc SCTransform v2 (Seaurat), followed by data integration across behavioral groups. Graph-based clustering was performed in PCA space. Cell-type identities were assigned by integration with 50,000 hippocampal and cortical cells from the Allen Brain Atlas (Yao et al., 2021) using query-based annotation.

Differential gene expression was assessed with the Wilcoxon rank-sum test [Seurat FindMarkers() and FindAllMarkers()]. Comparisons were made (1) between trained and untrained nuclei within each cell type and (2) between *eYFP^+^* (>0 expression of Gt(ROSA)26Sor mRNA) and *eYFP^−^* nuclei within hippocampal clusters (CA1, CA2, CA3, DG, inhibitory, astrocyte, oligodendrocyte). Genes with FDR < 0.05 were considered significant. Gene Ontology enrichment analyses were performed as described for spatial transcriptomics above.

## Data Availability

The original contributions presented in the study are publicly available. This data can be found here: Spatial Transcriptomic s– GSE330153, https://www.ncbi.nlm.nih.gov/geo/query/acc.cgi?acc=GSE330153; Single Nuclei – GSE330161, https://www.ncbi.nlm.nih.gov/geo/query/acc.cgi?acc=GSE330161.
