# Supplementary material for: Transcriptomic signatures of hippocampal active place avoidance memory maintenance
Source: Front Cell Neurosci. 2026 May 18;20:1769317. doi: 10.3389/fncel.2026.1769317 (PMC13222796; doi:10.3389/fncel.2026.1769317)
Supplement: Supplementary file 1 [file Data_Sheet_1.pdf]

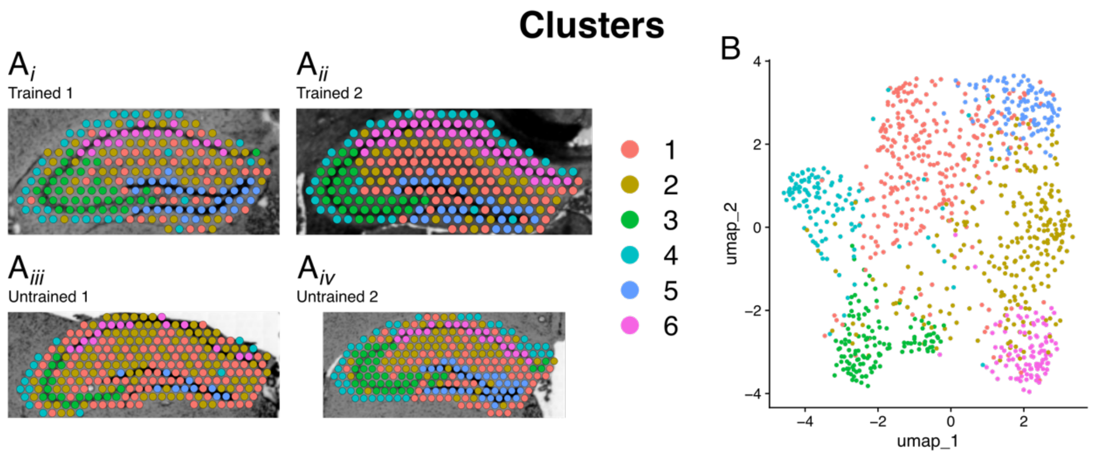

Clusters
A_i
Trained 1
A_ii
Trained 2
A_iii
Untrained 1
A_iv
Untrained 2
1
2
3
4
5
6
B
umap_2
umap_1

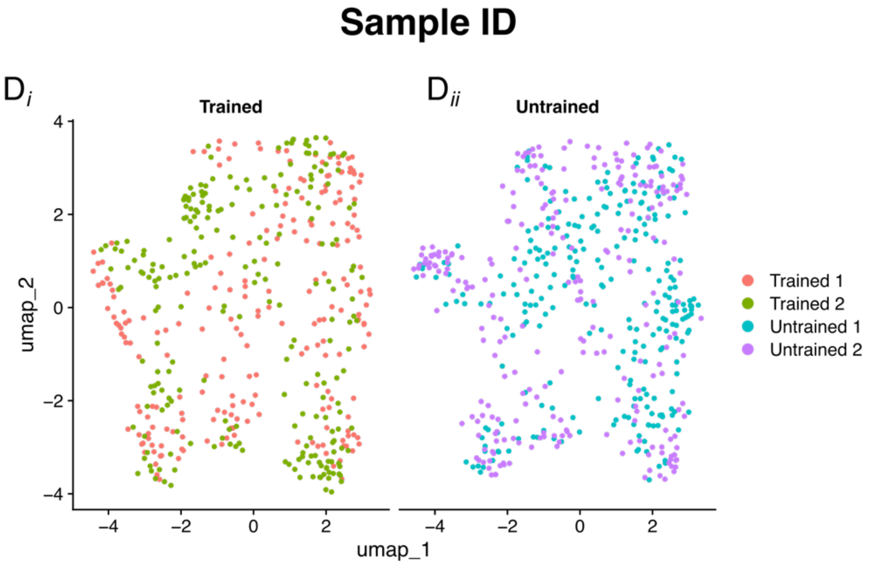

Sample ID
D_i
Trained
D_ii
Untrained
umap_2
umap_1
Trained 1
Trained 2
Untrained 1
Untrained 2

Figure 1. Clustering and cell-type annotation reflect anatomical boundaries of the hippocampal subregions.

(A_i-A_{iv}, B) Spatial transcriptomics map **(A_i-A_{iv})** and UMAP plot **(B)** of all hippocampal spots from trained and untrained mice (n = 2 per group). Discrete color scale displays unsupervised graph-based clustering of hippocampal spots. Unbiased clustering groups spots along anatomical boundaries reflecting natural transcriptomic differences between morphologically distinct regions in the hippocampus. **(C_i-C_{ii})** UMAP plots split along behavioral conditions demonstrates the relative distribution of spatial transcriptomic spots from each sample and their suitable computational integration.

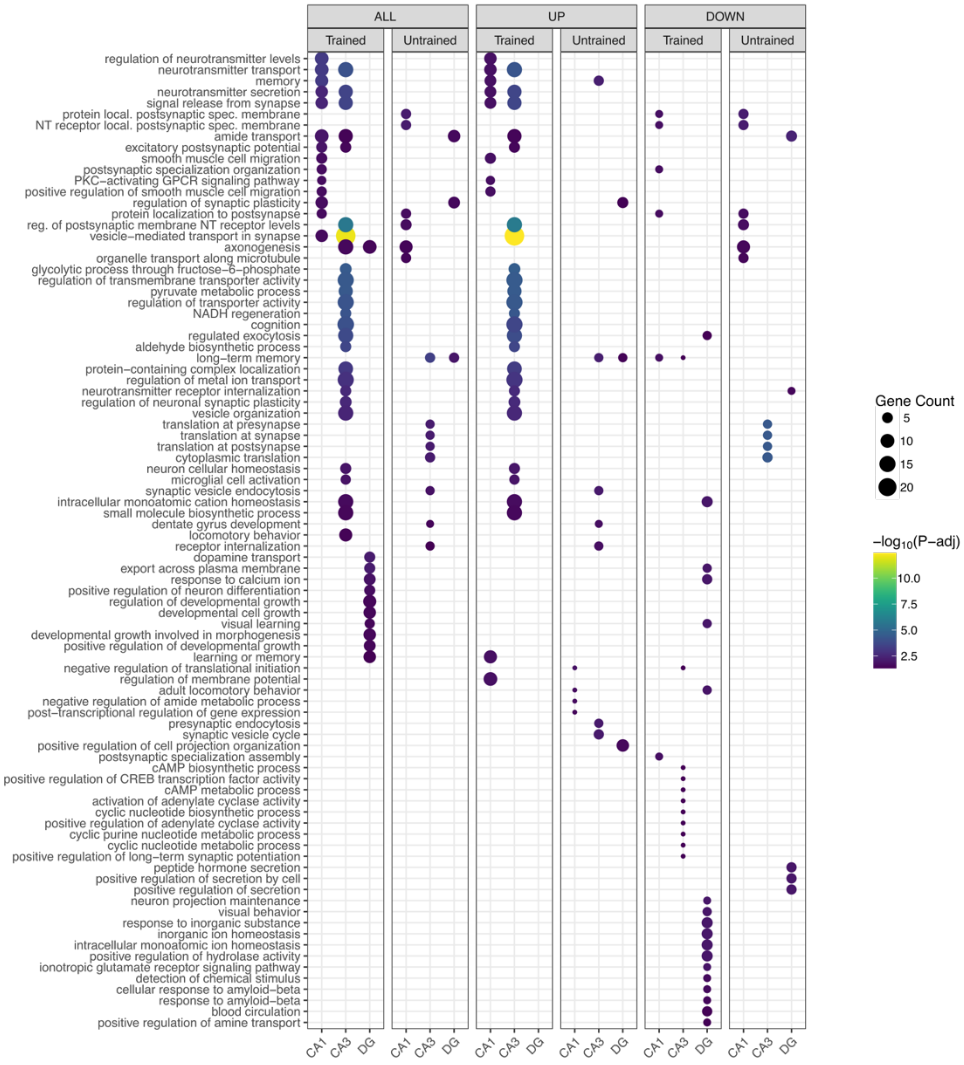
ALL
UP
DOWN
Trained
Untrained
regulation of neurotransmitter levels
neurotransmitter transport
memory
neurotransmitter secretion
signal release from synapse
protein local. postsynaptic spec. membrane
NT receptor local. postsynaptic spec. membrane
amide transport
excitatory postsynaptic potential
smooth muscle cell migration
postsynaptic specialization organization
PKC−activating GPCR signaling pathway
positive regulation of smooth muscle cell migration
regulation of synaptic plasticity
protein localization to postsynapse
reg. of postsynaptic membrane NT receptor levels
vesicle−mediated transport in synapse
axonogenesis
organelle transport along microtubule
glycolytic process through fructose−6−phosphate
regulation of transmembrane transporter activity
pyruvate metabolic process
regulation of transporter activity
NADH regeneration
cognition
regulated exocytosis
aldehyde biosynthetic process
long−term memory
protein−containing complex localization
regulation of metal ion transport
neurotransmitter receptor internalization
regulation of neuronal synaptic plasticity
vesicle organization
translation at presynapse
translation at synapse
translation at postsynapse
cytoplasmic translation
neuron cellular homeostasis
microglial cell activation
synaptic vesicle endocytosis
intracellular monoatomic cation homeostasis
small molecule biosynthetic process
dentate gyrus development
locomotory behavior
receptor internalization
dopamine transport
export across plasma membrane
response to calcium ion
positive regulation of neuron differentiation
regulation of developmental growth
developmental cell growth
visual learning
developmental growth involved in morphogenesis
positive regulation of developmental growth
learning or memory
negative regulation of translational initiation
regulation of membrane potential
adult locomotory behavior
negative regulation of amide metabolic process
post−transcriptional regulation of gene expression
presynaptic endocytosis
synaptic vesicle cycle
positive regulation of cell projection organization
postsynaptic specialization assembly
cAMP biosynthetic process
positive regulation of CREB transcription factor activity
cAMP metabolic process
activation of adenylate cyclase activity
cyclic nucleotide biosynthetic process
positive regulation of adenylate cyclase activity
cyclic purine nucleotide metabolic process
cyclic nucleotide metabolic process
positive regulation of long−term synaptic potentiation
peptide hormone secretion
positive regulation of secretion by cell
positive regulation of secretion
neuron projection maintenance
visual behavior
response to inorganic substance
inorganic ion homeostasis
intracellular monoatomic ion homeostasis
positive regulation of hydrolase activity
ionotropic glutamate receptor signaling pathway
detection of chemical stimulus
cellular response to amyloid−beta
response to amyloid−beta
blood circulation
positive regulation of amine transport
CA1 CA3 DG
Gene Count
5
10
15
20
−log10(P−adj)
10.0
7.5
5.0
2.5

Figure 2. DEGs detected between trained and untrained animals within hippocampal subregions using spatial transcriptomics are enriched with genes involved in synaptic plasticity processes.

Regional enrichment of biological processes detected amongst all differentially expressed genes (left) and stratified by up- (middle) and down-regulated (right) differentially expressed genes. Dot color reflects the statistical significance (-$\log_{10}$(FDR)) of the biological process enrichment. Dot size reflects the number of detected differentially expressed genes mapped to the genes involved in a given biological process.

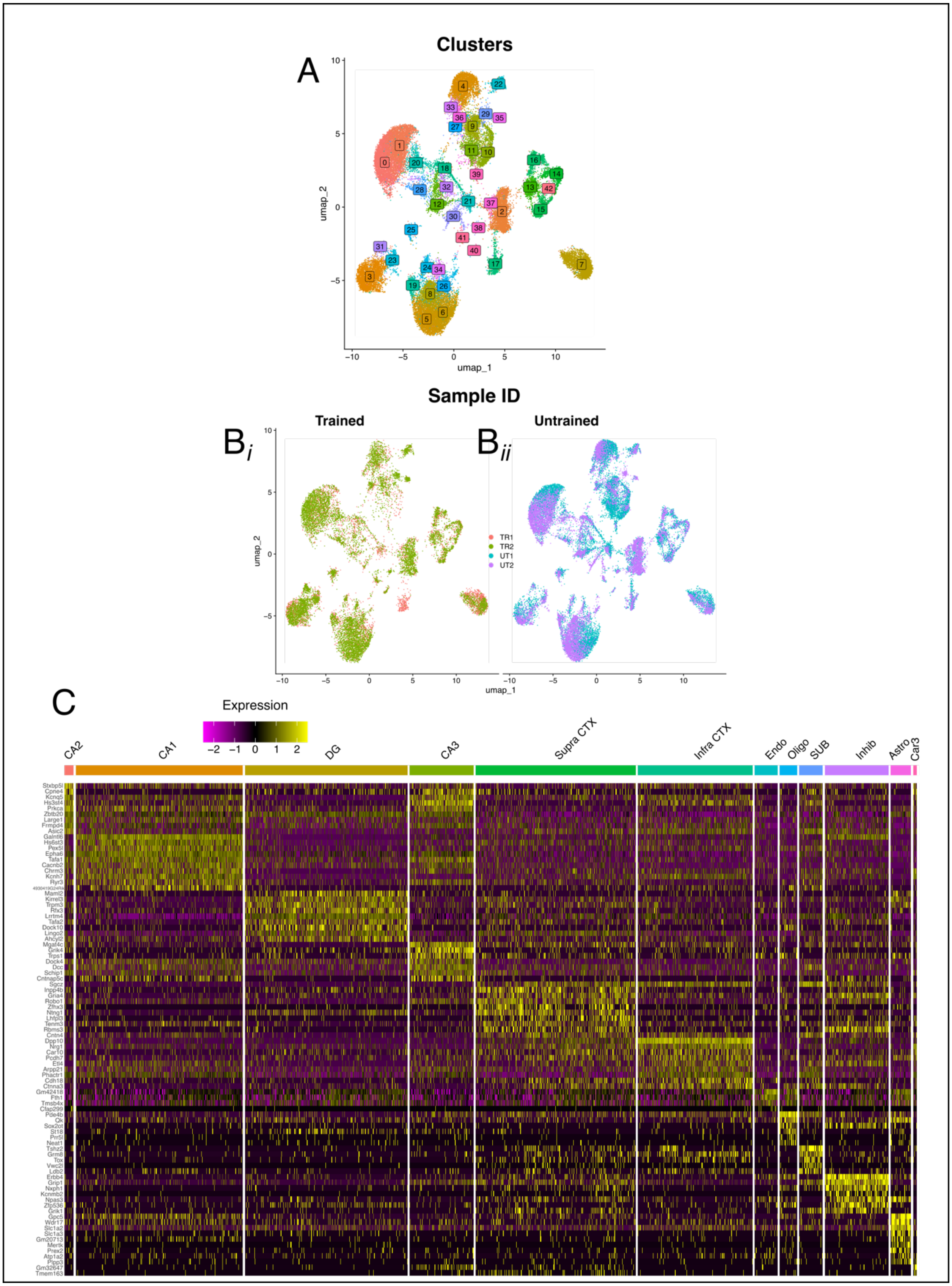
Clusters
A
umap_1
umap_2
Sample ID
Trained
Untrained
Bi
Bii
TR1
TR2
UT1
UT2
C
Expression
CA2
CA1
DG
CA3
Supra CTX
Infra CTX
Endo
Oligo
SUB
Inhib
Astro
Car3

Figure 3. Clustering of nuclei reflects hippocampal subregional cell-types.

(A) UMAP plot of clustered hippocampal nuclei. Discrete color scale displays unsupervised graph-based clustering (resolution = 1.5) separating nuclei into 42 distinct clusters. **(B_i, B_{ii})** UMAP plots split along behavioral conditions demonstrates the relative distribution of sequenced nuclei from each sample and their suitable computational integration. **(C)** Gene expression heatmap with each row showing the relative expression of one of the top 10 significant differentially expressed genes marking each cell-type cluster. Columns represent a random subset of 10,000 nuclei grouped by their cell type clustering and arranged by a hierarchical clustering dendrogram (not shown). Cells are colored in a continuous scale by the normalized expression of each gene in each sampled nucleus.

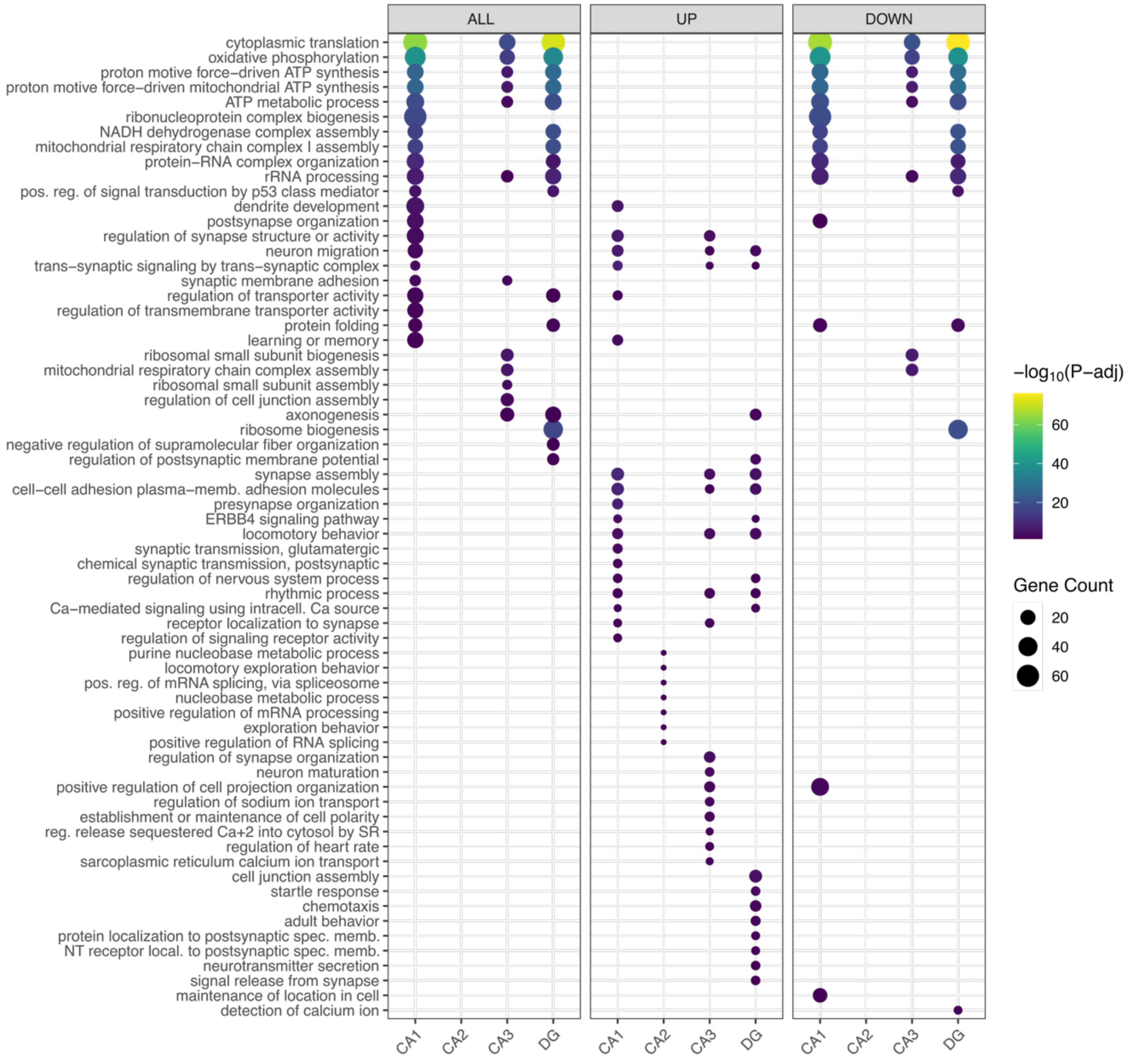

ALL
UP
DOWN
cytoplasmic translation
oxidative phosphorylation
proton motive force−driven ATP synthesis
proton motive force−driven mitochondrial ATP synthesis
ATP metabolic process
ribonucleoprotein complex biogenesis
NADH dehydrogenase complex assembly
mitochondrial respiratory chain complex I assembly
protein−RNA complex organization
rRNA processing
pos. reg. of signal transduction by p53 class mediator
dendrite development
postsynapse organization
regulation of synapse structure or activity
neuron migration
trans−synaptic signaling by trans−synaptic complex
synaptic membrane adhesion
regulation of transporter activity
regulation of transmembrane transporter activity
protein folding
learning or memory
ribosomal small subunit biogenesis
mitochondrial respiratory chain complex assembly
ribosomal small subunit assembly
regulation of cell junction assembly
axonogenesis
ribosome biogenesis
negative regulation of supramolecular fiber organization
regulation of postsynaptic membrane potential
synapse assembly
cell−cell adhesion plasma−memb. adhesion molecules
presynapse organization
ERBB4 signaling pathway
locomotory behavior
synaptic transmission, glutamatergic
chemical synaptic transmission, postsynaptic
regulation of nervous system process
rhythmic process
Ca−mediated signaling using intracell. Ca source
receptor localization to synapse
regulation of signaling receptor activity
purine nucleobase metabolic process
locomotory exploration behavior
pos. reg. of mRNA splicing, via spliceosome
nucleobase metabolic process
positive regulation of mRNA processing
exploration behavior
positive regulation of RNA splicing
regulation of synapse organization
neuron maturation
positive regulation of cell projection organization
regulation of sodium ion transport
establishment or maintenance of cell polarity
reg. release sequestered Ca+2 into cytosol by SR
regulation of heart rate
sarcoplasmic reticulum calcium ion transport
cell junction assembly
startle response
chemotaxis
adult behavior
protein localization to postsynaptic spec. memb.
NT receptor local. to postsynaptic spec. memb.
neurotransmitter secretion
signal release from synapse
maintenance of location in cell
detection of calcium ion
CA1
CA2
CA3
DG
−log10(P−adj)
60
40
20
Gene Count
20
40
60

Figure 4. DEGs detected between trained and untrained animals within hippocampal cell-type clusters are enriched with genes involved in translation and ATP synthesis.

Regional enrichment of biological processes detected amongst all differentially expressed genes (left) and stratified by up- (middle) and down-regulated (right) differentially expressed genes. Dot color reflects the statistical significance ($-\log_{10}$(FDR)) of the biological process enrichment. Dot size reflects the number of detected differentially expressed genes mapped to the genes involved in a given biological process.

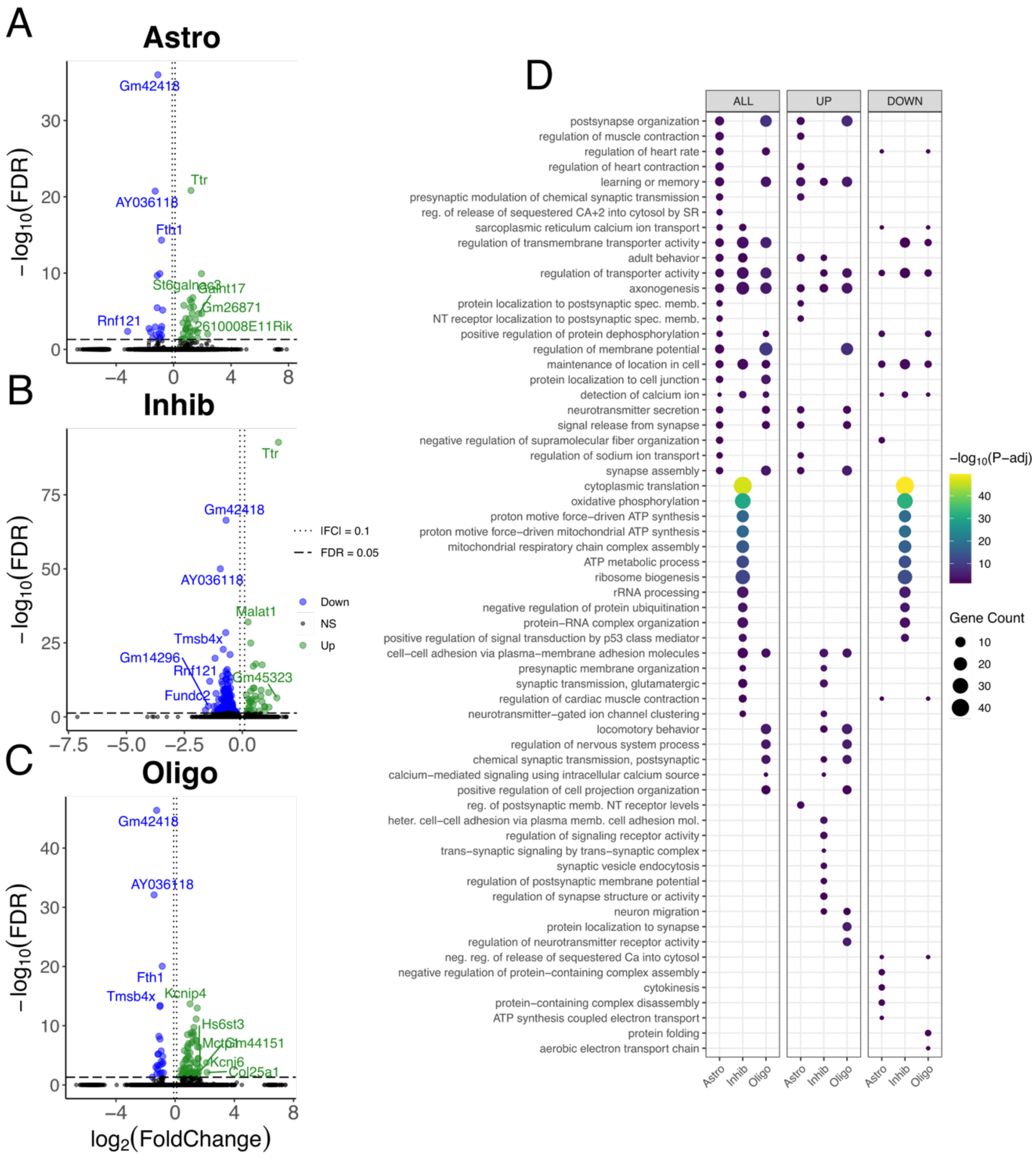
A
Astro
Gm42418
AY036118
Fth1
Ttr
St6galnac3
Galnt17
Gm26871
2610008E11Rik
Rnf121
−log10(FDR)
B
Inhib
Ttr
Gm42418
AY036118
Malat1
Tmsb4x
Gm14296
Rnf121
Gm45323
Fundc2
lFCl = 0.1
FDR = 0.05
Down
NS
Up
C
Oligo
Gm42418
AY036118
Fth1
Tmsb4x
Kcnip4
Hs6st3
Mctp1
Gm44151
Kcnj6
Col25a1
log2(FoldChange)
D
ALL
UP
DOWN
postsynapse organization
regulation of muscle contraction
regulation of heart rate
regulation of heart contraction
learning or memory
presynaptic modulation of chemical synaptic transmission
reg. of release of sequestered CA+2 into cytosol by SR
sarcoplasmic reticulum calcium ion transport
regulation of transmembrane transporter activity
adult behavior
regulation of transporter activity
axonogenesis
protein localization to postsynaptic spec. memb.
NT receptor localization to postsynaptic spec. memb.
positive regulation of protein dephosphorylation
regulation of membrane potential
maintenance of location in cell
protein localization to cell junction
detection of calcium ion
neurotransmitter secretion
signal release from synapse
negative regulation of supramolecular fiber organization
regulation of sodium ion transport
synapse assembly
cytoplasmic translation
oxidative phosphorylation
proton motive force−driven ATP synthesis
proton motive force−driven mitochondrial ATP synthesis
mitochondrial respiratory chain complex assembly
ATP metabolic process
ribosome biogenesis
rRNA processing
negative regulation of protein ubiquitination
protein−RNA complex organization
positive regulation of signal transduction by p53 class mediator
cell−cell adhesion via plasma−membrane adhesion molecules
presynaptic membrane organization
synaptic transmission, glutamatergic
regulation of cardiac muscle contraction
neurotransmitter−gated ion channel clustering
locomotory behavior
regulation of nervous system process
chemical synaptic transmission, postsynaptic
calcium−mediated signaling using intracellular calcium source
positive regulation of cell projection organization
reg. of postsynaptic memb. NT receptor levels
heter. cell−cell adhesion via plasma memb. cell adhesion mol.
regulation of signaling receptor activity
trans−synaptic signaling by trans−synaptic complex
synaptic vesicle endocytosis
regulation of postsynaptic membrane potential
regulation of synapse structure or activity
neuron migration
protein localization to synapse
regulation of neurotransmitter receptor activity
neg. reg. of release of sequestered Ca into cytosol
negative regulation of protein−containing complex assembly
cytokinesis
protein−containing complex disassembly
ATP synthesis coupled electron transport
protein folding
aerobic electron transport chain
Astro
Inhib
Oligo
−log10(P−adj)
Gene Count

Figure 5. DEGs detected between trained and untrained animals within astrocyte, oligodendrocyte, and inhibitory interneuron clusters demonstrate enrichment of biological processes similar to those seen in hippocampal neuronal clusters.

(A-C) Volcano plot of differentially expressed genes between inhibitory interneuron **(A)**, astrocyte **(B)**, and oligodendrocyte **(C)** nuclei clusters between trained and untrained mice (n = 2 male per group). In the analysis of inhibitory interneuron nuclei comparing trained and untrained conditions, 38 genes were upregulated and 237 were downregulated in the trained condition. 35 genes were upregulated and 19 were downregulated in the astrocyte nuclei, and 77 genes were upregulated and 23 were downregulated in the oligodendrocyte nuclei **(D)** Regional enrichment of biological processes detected amongst all differentially expressed genes (left) and stratified by up- (middle) and down-regulated (right) differentially expressed genes. Dot color reflects the statistical significance ($-\log_{10}(FDR)$) of the biological process enrichment. Dot size reflects the number of detected differentially expressed genes mapped to the genes involved in a given biological process.

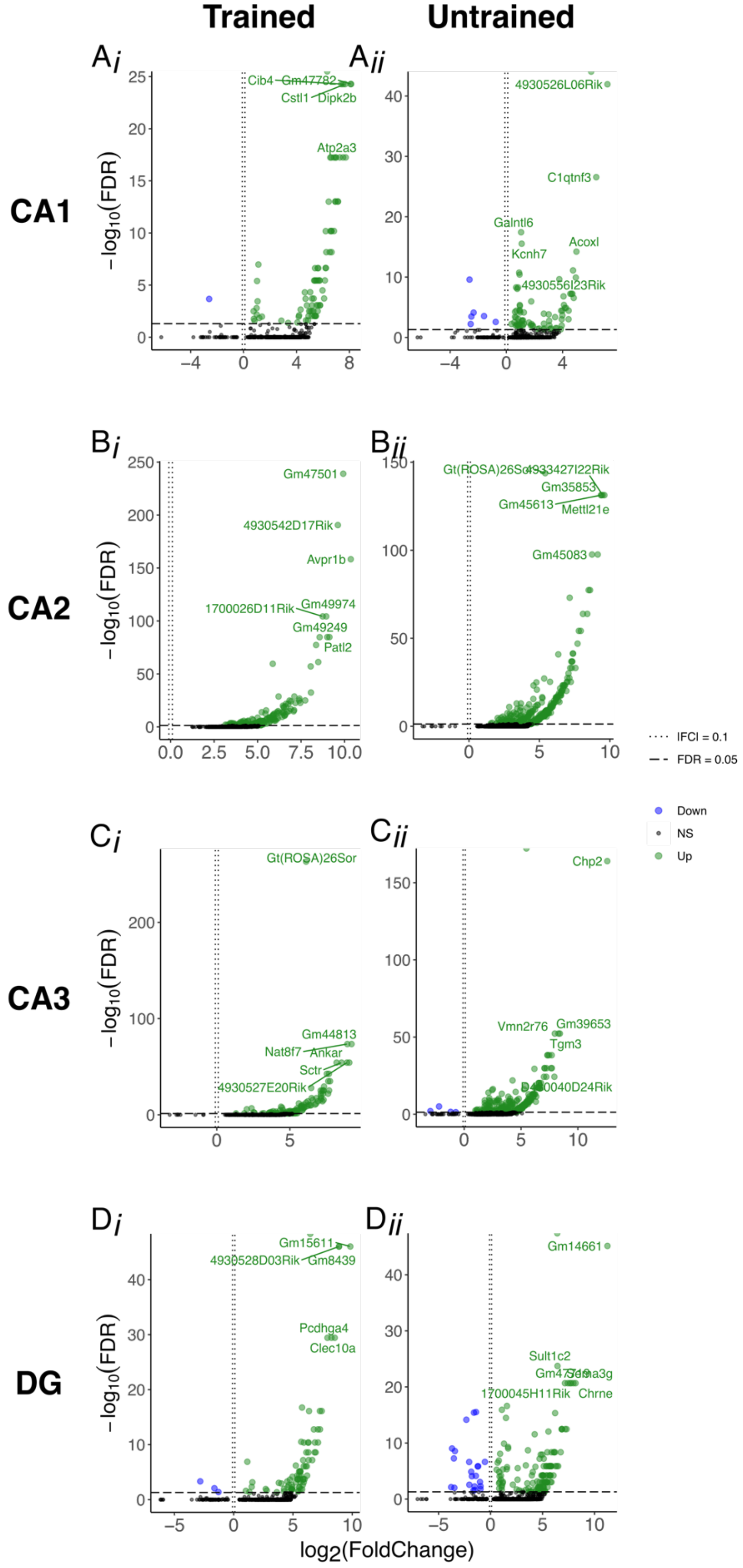

Trained
Untrained
CA1
CA2
CA3
DG
Ai
Aii
Bi
Bii
Ci
Cii
Di
Dii
−log10(FDR)
log2(FoldChange)
Cib4
Gm47782
Cstl1
Dipk2b
Atp2a3
4930526L06Rik
C1qtnf3
Galntl6
Kcnh7
Acoxl
4930556I23Rik
Gm47501
4930542D17Rik
Avpr1b
1700026D11Rik
Gm49974
Gm49249
Patl2
Gt(ROSA)26Sor
4933427I22Rik
Gm35853
Gm45613
Mettl21e
Gm45083
lFCl = 0.1
FDR = 0.05
Down
NS
Up
Gm44813
Nat8f7
Ankar
Sctr
4930527E20Rik
Chp2
Vmn2r76
Gm39653
Tgm3
Gm15611
4930528D03Rik
Gm8439
Pcdhga4
Clec10a
Gm14661
Sult1c2
1700045H11Rik
Chrne

Figure 6. eYFP-positive nuclei in the trained sample are marked by genes involved in calcium-driven signal transduction and membrane excitability.

(A_i –D_{ii}) Volcano plots of differential gene expression marking eYFP+ nuclei in the trained condition for CA1 nuclei **(A_i)**, CA2 nuclei **(B_i)**, CA3 nuclei **(C_i)**, and DG nuclei **(D_i). (A_{ii} – D_{ii})** Volcano plots of differential gene expression marking eYFP+ nuclei in the untrained condition for CA1 nuclei **(A_{ii})**, CA2 nuclei **(B_{ii})**, CA3 nuclei **(C_{ii})**, and DG nuclei **(D_{ii}).**
